# Influence of Mn Ions’ Insertion in Pseudo-Tetragonal Phased CaBi_4_Ti_4_O_15_-Based Ceramics for Highly Efficient Energy Storage Devices and High-Temperature Piezoelectric Applications

**DOI:** 10.3390/ijms232112723

**Published:** 2022-10-22

**Authors:** Ahmad Hussain, Nawishta Jabeen, Najam Ul Hassan, Altaf Ur Rahman, Muhammad Usman Khan, Adeela Naz, El Sayed Yousef

**Affiliations:** 1 Department of Physics, The University of Lahore, Sargodha Campus, Sargodha 40100, Pakistan; 2 Department of Physics, Fatima Jinnah Women University Rawalpindi, Rawalpindi 46000, Pakistan; 3 Department of Physics, Division of Science and Technology, University of Education, Lahore 54000, Pakistan; 4 Department of Physics, Riphah International University, Lahore 54000, Pakistan; 5 Research Center for Advanced Material Science (RCAMS), King Khalid University, P.O. Box 9004, Abha 61413, Saudi Arabia; 6 Physics Department, Faculty of Science, King Khalid University, P.O. Box 9004, Abha 61413, Saudi Arabia

**Keywords:** BLSF’s, pseudo-tetragonal, CaBi_4_Ti_4_O_15_, MnO_2_, ferroelectric, piezoelectric, energy storage

## Abstract

In the present era of advanced technology, the surge for suitable multifunctional materials capable of operating above 300 °C has increased for the utilization of high-temperature piezoelectric devices. For this purpose, a pseudo-tetragonal phased CaBi_4_Ti_3.98_ (Nb_0.5_Fe_0.5_)_0.02_O_15_:*x*wt%MnO_2_ (CBTNF:*x*Mn), with *x* = 0–0.20, ceramic system has been engineered for the investigation of structural, ferroelectric, dielectric and high-temperature-dependent piezoelectric properties. XRD analysis confirms that low-content Mn-ion insertion at the lattice sites of CBTNF does not distort the pseudo-tetragonal phase lattice of CBTNF:*x*Mn ceramics, but enhances the functional behavior of the ceramic system, specifically at *x* = 0.15 wt%Mn. Compared to pure CBT and CBTNF ceramics, CBTNF:0.15Mn has demonstrated a highly dense relative density (~96%), a saturated polarization (*P_S_*) of 15.89 µC/cm^2^, a storage energy density (*W_ST_*) of ~1.82 J/cm^3^, an energy-conversion efficiency (*ƞ*) of ~51% and an upgraded piezoelectric behavior (*d_33_*) of 27.1 pC/N at room temperature. Sharp temperature-dependent dielectric constant (*ε_r_*) peaks display the solid ferroelectric behavior of the CBTNF:0.15Mn sample with a Curie temperature (*T_C_*) of 766 °C. The thermally stable piezoelectric performance of the CBTNF:0.15Mn ceramic was observed at 600 °C, with just a 0.8% *d_33_* loss (25 pC/N). The achieved results signify that multi-valence Mn ions have effectively intercalated at the lattice sites of the pseudo-tetragonal phased CBTNF counterpart and enhanced the multifunctional properties of the ceramic system, proving it to be a durable contender for utilization in energy-storage applications and stable high-temperature piezoelectric applications.

## 1. Introduction

Sensors, actuators and transducers are the device-based practical applications of piezoelectric materials needed to operate at high temperatures. These applications are capable of performing as ultrasound transducers for non-destructive evaluation and non-destructive testing in high-temperature environments [1,2,3]. Hence, the materials capable of being utilized in these applications have achieved significant attention, especially in the field of space exploration (solar system), where the working conditions remain very harsh, such as temperatures of 460 °C (Venus) [4,5]. Nowadays, dielectric capacitors have also attracted much consideration in the form of ceramics and thin films to demonstrate rapid charge–discharge rates and an improved energy-storage density. Such fabricated materials have shown a tendency to be utilized in pulsed power and in powering portable/wearable electronic applications. Yang et al. synthesized the Mn-doped 0.97(0.93Na_0.5_Bi_0.5_TiO_3_-0.07BaTiO_3_)-0.03BiFeO_3_ flexible thin film, which demonstrated high efficiency (*η* ≈ 64.4%) [6]. Lv et al. synthesized the multilayer structure based (Na_0.8_K_0.2_)_0.5_Bi_0.5_TiO_3_ and Ba_0.5_Sr_0.5_ (Ti_0.97_Mn_0.03_)O_3_ thin film, which showed a high efficiency (*η*) of 68% [7].

The materials of the Aurivillius-type family, also known as bismuth-layered structure ferroelectrics (BLSFs) with a (Bi_2_O_2_)^2+^ (A_m-1_B_m_O_3m+1_)^2−^ general formula, have been proposed to be excellent candidates for use in such high-temperature applications [8,9,10]. Such materials naturally possess a high Curie temperature (*T_C_*), which results in high ferro–paraelectric phase-transition temperatures and shows the thermally stable piezoelectric response [11,12]. Calcium bismuth titanate, CaBi_4_Ti_4_O_15_ (CBT) with *m* = 4 is a widely studied BLSFs material, which possesses orthorhombic symmetry with the *A_21_am* space group at room temperature [11,13,14,15]. CBT has a high *T_C_* of ~780 °C, but because of its layered structure which limits the material-transportation properties during sintering progress and its spontaneous polarization (along *a-b* plane) value to a low piezoelectric coefficient (*d_33_*) of ~8 pC/N, its applications are restricted [16,17]. There exist several research reports where scientists have reported their work on improving the values of remnant polarization (*P_r_*), *d_33_* and *T_C_* by modifying fabrication techniques or by doping the ions at A and B sites of CBT. Recently, Wu et al. reported the fabrication of Gd/Mn co-coped CaBi_4_Ti_4_O_15_ ceramics, where a high *dc* conduction activation energy value of 1.87 eV was reported [18]. Xi et al. fabricated a Ca_0.85_ (LiCe)_0.075_Bi_4_Ti_4−*x*_Zn*x*O_15_ ceramic system and reported a high *d_33_* of 20 pC/N [13]. Xiao et al. reported the fabrication of a Ca_1−x_ (Li, Ho)_x/2_Bi_4_Ti_4_O_15_ ceramic system via a conventional solid-state reaction method, where a high resistivity of 4.51 × 10^11^ Ω cm was reported with an improved *T_C_* of 814 °C [19]. Liu et al. synthesized the CaBi_4_Ti_4−*x*_(Nb_1/2_Fe_1/2_)*_x_*O_15_ ceramic system with a low dielectric loss (tanδ) of 5.9% at 400 °C [14]. This achieved result of low dielectric loss is the reason for highly dense ceramic, which makes this synthesized scheme a strong candidate for further exploration.

MnO_2_ is an extensively explored material, owing to its natural abundance, low cost and low toxicity [20,21]. Moreover, the multi-valence nature of manganese (Mn^2+^, Mn^3+^ and Mn^4+^) ions, along with the various allotropic forms, have made MnO_2_ a strong candidate for the improvement of dielectric, ferroelectric and capacitive properties as a dopant/additive of the materials [22]. Recently, Hussain et al. reported the influence of Mn ions’ intercalation at the morphotropic phase boundary of Bi_0.5_ (K_0.2_Na_0.8_)_0.5_TiO_3_ as a ceramic with improved ferroelectric, piezoelectric and dielectric properties [23]. Yang et al. reported the fabrication of a flexible (Mn,Ti)-co-doped multiferroic BiFeO_3_ film capacitor, with a remanent polarization of 66 μC/cm^2^ [24]. Similarly, Jabeen et al. reported the synthesis of a Ca_0.60_ (Li_0.5_Bi_0.5_)_0.40_Bi_2_Nb_2_O_9_:*x*wt%MnO_2_ complex ceramic scheme, with an improvement in the ferroelectric and piezoelectric properties of the ceramic system [12]. Keeping in view this idea, as CaBi_4_Ti_4-*x*_ (Nb_0.5_Fe_0.5_)*_0.02_*O_15_ (CBTNF) usually shows a reduced dielectric loss at high temperatures with better piezoelectric properties, the combination of it with multi-valence additive MnO_2_ as CaBi_4_Ti_4-*x*_ (Nb_0.5_Fe_0.5_)*_0.02_*O_15_:xwt%MnO_2_ (CBTNF:xMn) with its new scheme needs to be explored. Here, it is expected that, during the high- temperature fabrication of CBTNF, the diffusion of Ca/Bi ions from the A site will be replaced by Mn^2+^/Mn^3+^ ions, while the evaporation of Ti ions will be occupied by Mn^4+^ ions. Such a diverse or unique approach can be helpful in exploring BLSFs as a step forward in practical applications. Moreover, the capacitive performance of the CBT-based ceramic is measured for the first time.

Herein, the influence of MnO_2_ as an additive is analyzed in a pseudo-tetragonal phased CBTNF system through the composition of CBTNF:*x*Mn with *x* = 0–0.2 to explore the structural, morphological, ferroelectric and piezoelectric measurements. For CBTNF:0.15Mn ceramic, the existence of a pseudo-tetragonal region, along with the influence of multi-valance Mn ions at the A site of CBTNF, have caused a strong multifunctional character with a high *P_r_* of ~9.8 µC/cm^2^, high *d_33_* of 27.1 pC/N and impedance of ~10^9^ Ω, which are much better than pure CBT or pure CBTNF ceramics.

## 2. Results and Discussion

**Figure 1:**
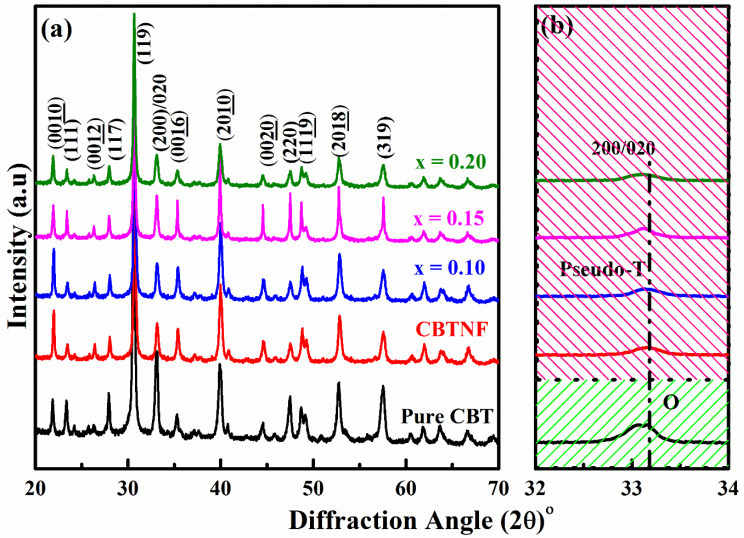
(**a**) XRD analysis of pure CBT (as reference) ceramic and CBTNF:*x*Mn ceramics with *x* = 0–0.20, (**b**) magnified image of XRD analysis (*2θ* = 32–34°) to confirm the existence of pseudo-tetragonal phase for CBTNF and CBTNF:*x*Mn (*x* = 0.10–0.20) ceramics.

The XRD study of pure single-phase CBT ceramic for comparison with pseudo-tetragonal phased CBTNF:*x*Mn (*x* = 0–0.20) ceramics are presented in Figure 1a. From the analysis, it can be observed that pure CBT ceramic maintained its layered Aurivillius-type single-phase structure with an orthorhombic structure (*A*21*am* space group), without the presence of impurities. The existence of a strong (119) diffraction peak at angle 30.8° for all compositions is the characteristic peak of the CBT-based ceramics [14]. XRD patterns have shown their consistency with the most intense (112 *m +* 1) diffraction peak of BLSFs [25,26]. An illustration of the pure single-phase CBT ceramic pattern at the bottom was made to compare the occurrence of structural distortions/transformations in other CBTNF:*x*Mn (*x* = 0–0.20 wt%) compositions. The pattern of the pure CBT ceramic is labeled *PDF #* 52–1640 [27]. The 200/020 peak splitting at angle 33.1° is a clear indication of the orthorhombic configuration of the single-phase CBT ceramic, although a syncretic 200/020 reflection for CBTNF:*x*Mn (*x* = 0–0.20) ceramics was observed. As high-temperature sintering was performed for the fabrication of ceramics, the A site vacancies varied due to the diffusion of Ca and Bi ions, causing the diminution of lattice parameters *a* and *c*, while *b* decreased. In Figure 1b, the tetragonality of the crystal is elaborated by the *a/b* ratio. The tetragonality increased with the variation of vacancies at the A site due to the evaporation of Bi at high-sintering temperatures, which has already been reported in articles belonging to the complex pseudo-tetragonal phase [28]. In this work, the tetragonality of the ceramics also increased under the influence of the varying content addition of Mn ions at the A site of CBTNF, after the evaporation of Bi ions due to high-sintering temperatures. This can be seen in other compositions of CBTNF:*x*Mn ceramics as well. All CBTNF:*x*Mn (*x* = 0–0.20) ceramics maintained their pseudo-tetragonal identity, verifying that Mn ions have efficaciously intercalated at the vacancies of parent CBTNF material, without damaging the structure as the content of additive Mn ions was kept very low.

**Figure 2:**
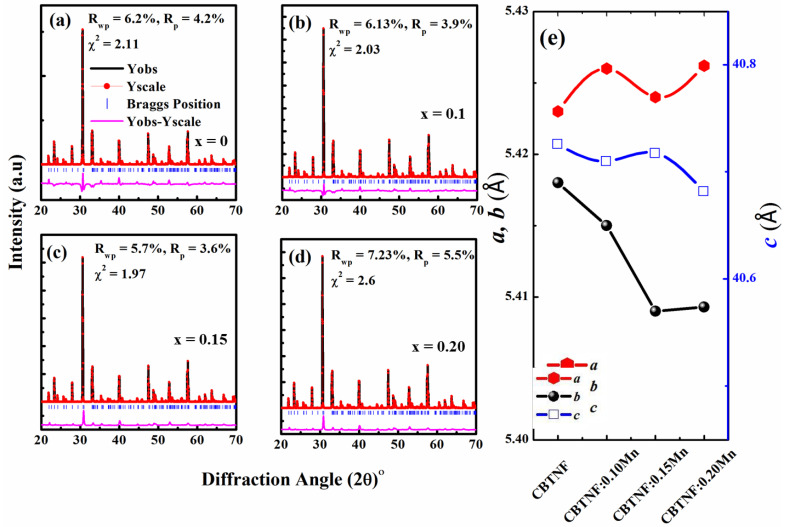
(**a**–**d**) Rietveld refinements for the CBTNF:xMn (*x* = 0.0, 0.1, 0.15 and 0.20) ceramics, (**e**) refined structural lattice parameters.

It is a common phenomenon that, during the doping or addition of the elements, the distortion of the lattice sites of the parent material occurs. However, the content of the Mn additive was consciously kept low to not disturb the lattice parameters of the CBTNF’s counterpart effectively. In order to observe the crystal structural conversion or crystal structure destruction, all the ceramic compositions were simulated. XRD Rietveld refinements of the pure CBTNF and CBTNF:xMn (*x* = 0.1, 0.15 and 0.20) ceramics were performed by employing the general diffraction-analysis software FULLPROF. Orthogonal symmetry with space group *A_21_am* was the best-fitting reference structure between the observed and calculated intensities. From Figure 2a–d, for all the refinements, excellent Rwp, Rp and χ^2^ refined factors were achieved, confirming the better agreement between the observed and calculated patterns. Obviously, there existed no large change in the lattice parameters (*a*, *b* and *c*), which is confirmation that Mn did not influence the pseudo-tetragonal phase of the CBTNF counterpart and maintained its identity (Figure 2e). 

**Figure 3:**
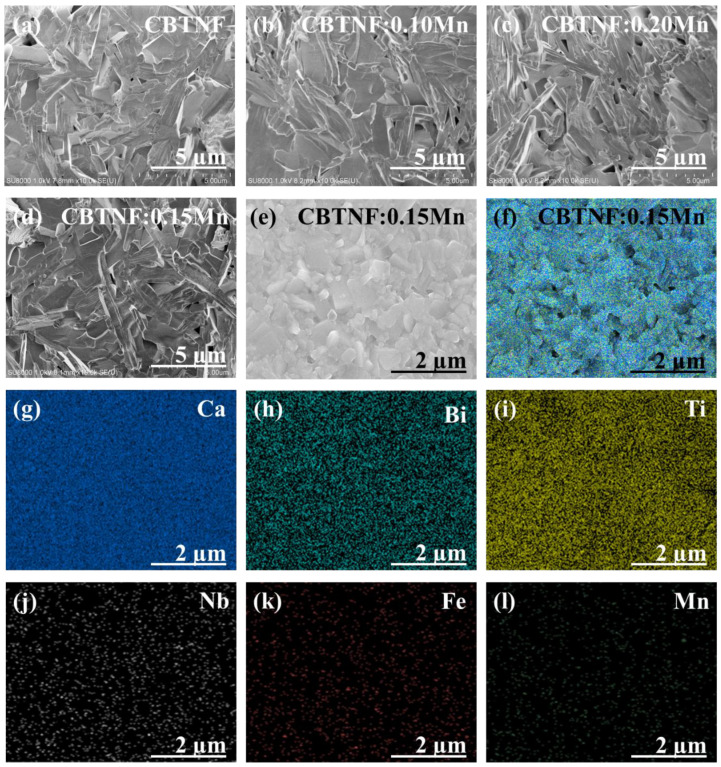
SEM cross-sectional analysis of ceramics for needle-like morphology (**a**) CBTNF, (**b**) CBTNF:0.10Mn, (**c**) CBTNF:0.20Mn, (**d**) CBTNF:0.15Mn, (**e**) surface FE-SEM analysis of CBTNF:0.15Mn ceramic, (**f**) colored elemental-dispersion analysis on the surface of CBTNF:0.15Mn ceramic, (**g**) Ca, (**h**) Bi, (**i**) Ti, (**j**) Nb, (**k**) Fe and (**l**) Mn.

For a perfect morphological comparison under the influence of an Mn additive, the cross-sectional SEM analysis of pure CBTNF (Figure 3a) is shown with the CBTNF:xMn (Figure 3b–d) ceramics. All samples confirmed the needle-like morphology. Morphological analysis of the CBTNF:0.15Mn ceramic sample and its two-dimensional (2D) elemental-mapping dispersion images are illustrated in Figure 3d–l. Figure 3d is a cross-sectional image of CBTNF:0.15Mn ceramic, and BLSFs are renowned for their plate-like surface and needle-like cross-section morphology [29]. Here, Figure 3d provides clear evidence of the needle-like morphology of the cross-sectional image of CBTNF:0.15Mn ceramic. Meanwhile, Figure 3e is the surface analysis for CBTNF:0.15Mn ceramic, where the regularity of the particle size < 2–3 μm can be seen. Cracks or holes in the ceramic were not observed, confirming the high density of the sample. Moreover, this fine-tuning of the particle size also verified the successful intercalation of the multi-valence Mn ions at the vacant sites of the parent CBTNF material without any kind of structural demolition, and it has reduced the lattice diffusivity. A high relative density of the sample of ~96% was measured. Figure 3f is the representation of the combined elemental mapping of all elements involved during synthesis and were assigned different colors to distinguish in CBTNF:0.15Mn ceramic. From the two-dimensional elemental-mapping images of Ca, Bi and Ti (Figure 3g–i), high degree of dispersion of elements was observed, which is totally dependent on the high contribution ratios of the constituent elements in CBTNF:0.15Mn. Meanwhile a minor degree of distribution was observed for Nb, Fe and Mn (Figure 3j–l), depending upon the less stoichiometric contribution of constituents in the CBTNF:0.15Mn chemical composition. There exists uniformity in the dispersion of additive Mn ions throughout the image, which is confirmation that Mn ions efficaciously intercalated at the vacant sites of the CBTNF material or accumulated at the grain boundaries. 

**Figure 4:**
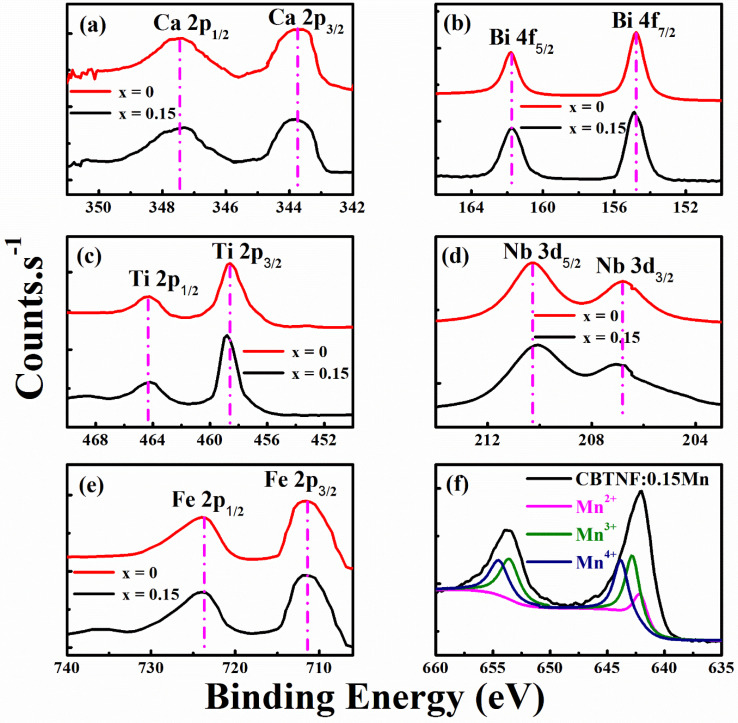
XPS spectral analysis of (**a**) Ca−2p, (**b**) Bi−4f, (**c**) Ti−2p, (**d**) Nb−3d, (**e**) Fe−2p, for pure CBTNF (black) and CBTNF:0.15Mn (red); fitted XPS analysis of multi-valance, (**f**) Mn ions in CBTNF:0.15Mn ceramic.

XPS analysis for the binding energy range of 0 to 750 eV was performed for the comparison study of pure CBTNF and CBTNF:0.15Mn ceramics. Additionally, the main course of the study was to analyze the presence of multi-valance manganese ions in the CBTNF:0.15Mn ceramic sample. Photoemissions of Ca-2p, Bi-4f, Ti-2p, Nb 3d and Fe-2p core levels are analyzed in Figure 4a–e. The XPS analysis of all the elements was confirmed by the National Institute of Standards and Technology XPS database. Binding energy peaks for Ca 2p_3/2_ and Ca 2p_1/2_ occurred at 347.48 eV and 343.7 eV, respectively, for both pure CBTNF and CBTNF:0.15Mn ceramics, confirming that the low content addition of Mn did not have an influence (Figure 4a). These binding energy values confirm the bivalent nature of Ca, consistent with the binding value of Ca-O in CaO [30]. In Figure 4b, it shown that the binding energies of Bi 4f_7/2_ for pure CBTNF was 154.78 eV, which is a bit of a shift for CBTNF:0.15Mn ceramic to 154.86 eV; meanwhile, for Bi 4 f_5/2_, the binding energy peak at 161.78 eV was consistent for both pure CBTNF and CBTNF:0.15Mn ceramics. This little shift of Bi 4f_7/2_ to higher values for CBTNF:0.15Mn ceramic can be attributed to the (Bi_2_O_2_)^2+^ layer, due to surrounding environment [31]. It is also evident from the XPS analysis that the Bi element does not work dominantly at the A site, which is also verifiable with the no peak shifting of the Ca element. In Figure 4c, the XPS spectra of Ti 2p of pure CBTNF and CBTNF:0.15Mn ceramics are shown. The peak for Ti 2p_3/2_ was located at a binding energy value of 458.6 eV, while the peak for Ti 2p_1/2_ was located at a binding energy value of 464.4 eV. These peak positions of Ti 2p_3/2_ and Ti 2p_1/2_ are the characteristic features of standard anatase phase TiO_2_ [32]. Moreover, the difference in binding energies between Ti 2p_3/2_ and Ti 2p_1/2_ (ΔBE = Ti 2p_3–2_ − Ti 2p_1/2_) was equal to 5.8 eV, which is associated with typical Ti^4+^–O bonding in TiO_2_ [33]. No obvious peak shifting was observed for Nb 3d_3/2_ at a binding energy of 206.8 eV and Nb 3d_5/2_ at a binding energy of 210.2 eV (Figure 4d). The peaks’ location along the binding energy values for Bi 4 f and Nb 3d (Figure 4b,d) of pure CBTNF and CBTNF:0.15Mn ceramics show the typical trivalent nature of bismuth and the pentavalent nature of niobium atoms [34]. The XPS spectra peaks of Fe 2p_3/2_ and Fe 2p_1/2_ for the pure CBTNF and CBTNF:0.15Mn ceramics are presented in Figure 4e, in which the peak of Fe 2p_3/2_ was located at the binding energy of 711.5 eV, while the peak of Fe 2p1/2 was located at 723.5 eV [35]. The difference in binding energies between Fe 2p_3/2_ and Fe 2p_1/2_ (ΔBE = Fe 2p_3–2_ − Fe 2p_1/2_) was equal to 11.8 eV. The XPS spectra for Mn 2p of CBTNF:0.15Mn ceramic is shown in Figure 4f, and the Mn 2p_3/2_ and Mn 2p_1/2_ peaks were located at the binding energies of 642.11 eV and 653.73 eV, respectively. The difference in binding energies between Mn 2p_3/2_ and Mn 2p_1/2_ (ΔBE = Mn 2p_3–2_ − Mn 2p_1/2_) was equal to 11.6 eV. Moreover, the Mn 2p_3/2_ and Mn 2p_1/2_ were fitted to visualize the multi-valence states of added Mn ions. From the simulation of the Mn 2p_3/2_ peak, three fitting peaks were located at binding energies 642.1, 642.75 and 644.6 eV, which were associated with Mn^2+^, Mn^3+^ and Mn^4+^, respectively. From Figure 4f, it is also evident that the surface area under the peak belonging to Mn^2+^ is much lesser than that of the other two peaks, confirming that a low amount of Mn^2+^ ions were generated compared to the Mn^3+^ and Mn^4+^ ions [12,36]. During the fabrication of ceramic materials, the sintering temperature plays a vital role in the production of multi-valence Mn ions [37]. Mn ions have the ability to intercalate with ease at the vacant lattice sites of the materials and generate easy domain wall mobility, causing the ferroelectric ceramics to demonstrate soft behavior and enhancing the ferroelectric and piezoelectric response [36].

**Figure 5:**
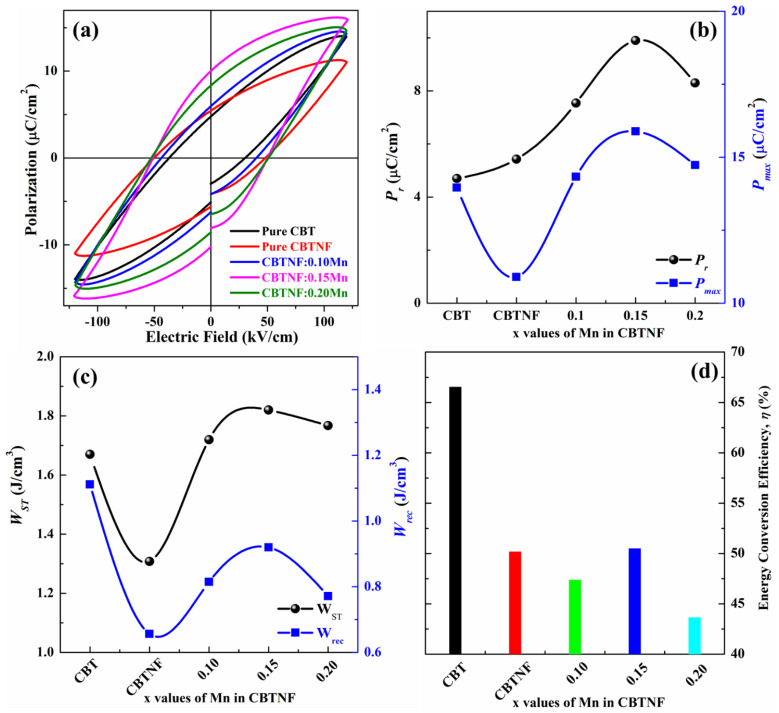
(**a**) *P-E* loops of pure CBT and CBTNF:*x*Mn (*x* = 0–0.20) ceramics, (**b**) *P_r_* and *P_max_* comparison values for pure CBT and *x*−valued CBTNF:*x*Mn (*x* = 0–0.20) ceramics, (**c**) *W_st_* and *W_rec_* values for pure CBT and *x*-valued CBTNF:*x*Mn (*x* = 0–0.20) ceramics, (**d**) energy conversion efficiency (*ƞ*) of the pure CBT and *x*−valued CBTNF:*x*Mn (*x* = 0–0.20) ceramics.

Ferroelectric analysis (*P-E* loops) of the pure CBT and CBTNF:*x*Mn (*x* = 0–0.20) ceramics is measured and presented in Figure 5a, where the highest saturated polarization (*P_S_*) of ~15.8 µC/cm^2^ was measured for CBTNF:0.15Mn. The measured *P_S_* value for CBTNF:0.15Mn was much improved compared to the pure CBT (*P_S_* ~13.8 µC/cm^2^) ceramic and pure CBTNF (*P_S_* ~10.8 µC/cm^2^) ceramic. A high electric field (120 kV/cm) was employed to all samples for these measurements. From Figure 5b, it can be stated that the value of *Pr* increased with the variation of additive Mn-ion content until the CBTNF:0.15Mn ceramic, which can be attributed to the presence of multi-valence Mn additives which acted as a soft agent for the CBTNF component, causing the improvement in the domain wall mobility, which allowed better broad switching within the ceramic [12,23]. Moreover, during the high-temperature sintering process of the ceramics, the highly voltaic nature of Bi was unavoidable, which resulted in the appearance of vacancies. Hence, for a charge-neutrality situation, oxygen ions started to evaporate as well, and these vacancies can be overcome by the intercalation of Mn ions to reduce the mobility defect and increment of ferroelectric properties [38]. Afterwards, for CBTNF:0.20Mn, the value of *P_S_* ~14.7 µC/cm^2^ started to decrease (Figure 5b). This decrement at CBTNF:0.20Mn is because the additive MnO_2_ content crossed the diffusible limit at the lattice sites, which is called the percolation threshold values, and the excessive Mn ions started to accumulate at the CBTNF/CBTNF grain boundaries. The conductivity of Mn ions will result in the creation of a leakage current and a decrement in ferroelectric properties [12].

Recently, the dielectric capacitor behavior of ceramics has achieved substantial attention in the electronic field of instant energy-storage applications, owing to their rapid charge–discharge time, high power density and exceptional chemical constancy. Recoverable energy density (*W_rec_*), stored energy density (*W_st_*) and energy-conversion efficiency (*ƞ*) calculations are achieved from the measured *P-E* loops, and the equations for the calculations are as follows [39]:

(1) $$W_{rec}=\int_{Pr}^{Pmax}Edp$$

(2) $$W_{st}=\int_{0}^{Pmax}Edp$$

(3) $$\eta =\frac{W_{rec}}{W_{st}}\times 100$$

where the values of *P_max_*, *P_r_* and *E* are achievable from the measured *P-E* loops. Figure 5c,d are the plots of calculated *W_rec_*, *W_st_* and *ƞ* versus the *x* values of Mn addition in CBTNF at 120 kV/cm. The values of stored energy density (*W_st_*) of all the samples were above 1.67 J/cm^3^ (1.67–1.82 J/cm^3^) and the energy-conversion efficiency (*ƞ*) was above 44% (44–63%). The highest *W_ST_* of 1.82 J/cm^3^ and *ƞ* of 51% were calculated for CBTNF:0.15Mn ceramic.

**Figure 6:**
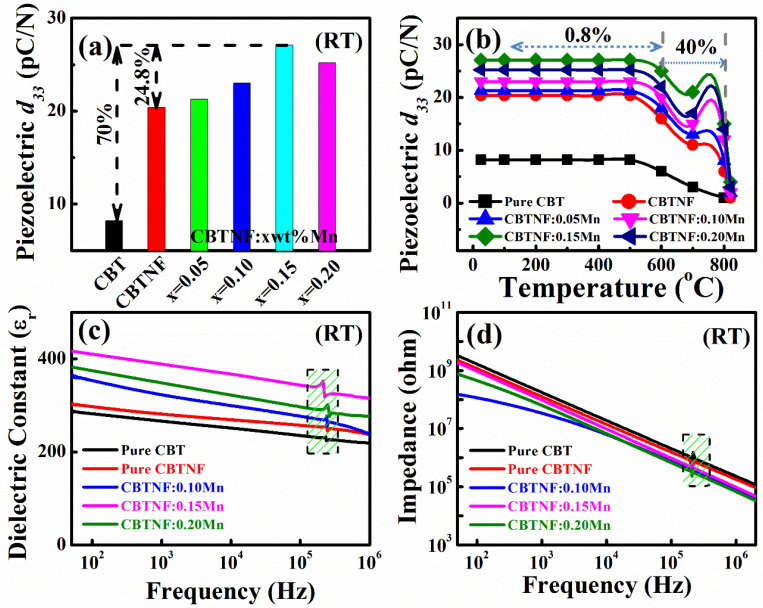
(**a**) Piezoelectric coefficient (*d_33_*) measurements of pure CBT and CBTNF:*x*Mn (*x* = 0–0.20) ceramics as a function of *x*wt%MnO_2_ addition in CBTNF samples, (**b**) thermal stability of piezoelectric coefficient (*d_33_*) for the temperature range 0–825 °C, (**c**) dielectric constant (*ε_r_*) versus frequency curves of pure CBT and CBTNF:xMn (*x* = 0–0.20) ceramics at room temperature, (**d**) impedance versus frequency plots of pure CBT and CBTNF:*x*Mn (*x* = 0–0.20) ceramics at room temperature.

The piezoelectric co-efficient (*d_33_*) values of pure CBT and CBTNF:xMn (*x* = 0–0.20) ceramics are presented in Figure 6a, measured at room temperature. The CBTNF:0.15Mn sample showed the strongest piezoelectric character with *d_33_* ~27.1 pC/N, much more improved than pure CBT (~ 8 pC/N) ceramic, with a 70% improvement and pure CBTNF (20 pC/N) ceramic with a 24.8% improvement. The thermal stability of the piezoelectric performance of the synthesized material under the influence of high temperatures was observed: the degradation of the piezoelectric properties in ceramic is observed and illustrated in Figure 6b, after annealing the ceramics at temperatures 25–825 °C for 30 min. The piezoelectric co-efficient of pure CBT degraded from 8 pC/N to 3 pC/N (at 700 °C) due to the high-temperature annealing effect. It reduced to 0 pC/N at 800 °C because the value of the Curie temperature of pure CBT was 780 °C, which had already passed. It is obvious that the Curie temperature is the limit where materials lose their piezoelectric properties completely with the ferroelectric-to-paraelectric transformation [40]. Similarly, the *d_33_* value of pure CBTNF remained much stronger (11 pC/N) at 700 °C than pure CBT. However, *d_33_* of CBTNF:0.15Mn showed the most stable character with 25 pC/N at 600 °C (0.8% loss of *d_33_*) and 21 pC/N at 700 °C (23% loss of *d_33_*). The applied electric field is the main reason for the polarization of the ceramics: this electric field creates the configuration of the ferroelectric domains within the CBTNF counterpart, and a liberated eccentric recompense of charges occurs at the CBTNF/CBTNF grain boundaries. During the annealing process, there exists a natural phenomenon within ceramics when materials transfer from an aligned domain configuration to a non-aligned domain configuration. This phenomenon is called back-switching at the diffusion temperature. The addition of Mn ions due to its multi-valence nature is capable of occupying the unbound misfits, which results in the development of electronic-charge dispersal at the grain boundaries, which reduce back-switching occurrence even at high-temperature annealing [38]. Herein, it can be observed that for CBTNF:0.20Mn ceramic, the *d_33_* value decreased to 25.2 pC/N (at room temperature) as the solubility limit of the Mn ions at vacant A and B sites in the CBTNF counterpart attained its maximum limit (percolation threshold). Later, ions will gather at the CBTNF/CBTNF grain boundaries, affecting the piezoelectric properties [41]. The frequency-dependent dielectric constant (*ε_r_*) plots of the fully poled CBT and CBTNF:*x*Mn (*x* = 0–0.20) samples are depicted in Figure 6c, where *ε_r_* of CBTNF:0.15Mn ceramic is at its maximum (*ε_r_* ~418) at a frequency of 100 Hz, which is better than all other compositions. For instance, all compositions of the ceramics were poled, causing the creation of dielectric anomalies along the thickness of the ceramic at certain frequencies (~212/225 kHz). Poled ceramics possess the novel capability to create deformation in their piezoelectric properties due to the influence of an applied 0.5 *AC* voltage, resulting in the formation of dielectric anomalies [42]. The impedance versus frequency plots of all fully poled ceramics is presented in Figure 6d, and materials belonging to the BLSF family are renowned for their high-resistance values. The impedance values of pure CBT (~4.1 × 10^9^ Ω) and CBTNF (~3.05 × 10^9^ Ω) ceramics were higher than all other compositions at a frequency 100 Hz. Mn ions caused the reduction in the impedance values of the materials, but this is a minute decrement (~1.2 × 10^9^ Ω for CBTNF:0.15Mn ceramic). Furthermore, the piezoelectric anomalies along the thickness of the impedance curves are consistent with the specific frequency (*ε*_r_ vs. *f* plot) detected because of piezoelectric (*d_33_*) influence [43].

**Figure 7:**
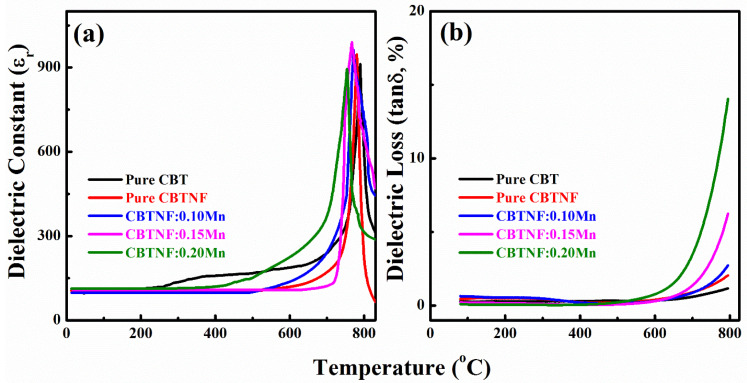
(**a**) Relative dielectric constant (*ε*_r_) as a function of temperature for pure CBT and CBTNF:*x*Mn (*x* = 0–0.20) ceramics, (**b**) dielectric loss (*tan δ*) as a function of temperature for pure CBT and CBTNF:*x*Mn (*x* = 0–0.20) ceramics.

The thermal stability of *d_33_* of poled ceramics is dependent on the values of the Curie temperature (*T_C_*) of the materials, where they not only lose their piezoelectric behavior, but also transform from the ferroelectric to the paraelectric phase. *T_C_* of the synthesized pure CBT and CBTNF:*x*Mn (*x* = 0–0.20) ceramics was taken at 100 kHz by temperature-dependent dielectric constant plots for a temperature range of 0 to 850 °C. Plots are presented in Figure 7a. The pure CBT showed the highest value of *T_C_* ~789 °C, consistent with previous reports. The *T_C_* values of pure CBTNF, CBTNF:0.1Mn, CBTNF:0.15Mn and CBTNF:0.2Mn ceramics were 779 °C, 770 °C, 766 °C and 753 °C, respectively. The attained values demonstrate the prominence of the fabricated material for utilization in various purpose devices. Frequency-dependent dielectric loss (*tanδ*) curves of the CBT and CBTNF:xMn (*x* = 0–0.20) samples are presented in Figure 7b, where it is evident that the dielectric loss is very low (less than 4%) for all compositions, even at 700 °C. This result reveals the high density of the ceramic materials, and confirms the successful incorporation of multi-valence Mn ions at the vacancies offered by CBTNF counterpart, devoid of generating any demolition of the structure.

## 3. Materials and Methods

Composition CaBi_4_Ti_4_O_15_ and CaBi_4_Ti_4-*x*_ (Nb_0.5_Fe_0.5_)*_0.02_*O_15_ ceramics were synthesized by a rapid liquid-sintering technique with a rapid increase and decrease in temperature > 20 °C/min to avoid the excessive diffusion of Bi ions and oxygen during the high-temperature sintering process. The high-purity raw powders of CaCO_3_ (99.9%, Aldrich, St. Louis, MO, USA), Bi_2_O_3_ (99.99%, Aladin, Shanghai, China), TiO_2_ (99.9%, Aldrich), Fe_2_O_3_ (99.9%, Aladin) and Nb_2_O_5_ (99.99%, Aladin) were weighed according to the desired stoichiometric ratio of CBT and CBTNF, mixed in a milling machine for 12 h in ethanol. Later on, the solution was dried at 100 °C overnight, calcined at 900 °C for 4 h, milled again for 12 h and dried at 100 °C. The dried powders of CBT and CBTNF were pelletized with a diameter of 7–11 mm under a hydrostatic pressure of 160 MPa and sintered at 1100 °C for 2–4 h. MnO_2_ (99.99%, Aladin) powder was stoichiometrically added in as a fabricated single-phased CBTNF powder, with a variation from 0.00 wt% to 0.20 wt% and a composition of CaBi_4_Ti_4-*x*_ (Nb_0.5_Fe_0.5_)*_0.02_*O_15_:*x*wt%MnO_2_ (CBTNF:*x*Mn). Powders were milled for 12 h and dried, and then polyvinyl butyral (PVB) was added as a binder to make the powder more dense. Lastly, the powders were pressed into 7–11 mm diameter disk ceramics under a hydrostatic pressure of 160 Mpa and sintered at 1100 °C for 4 h through a 20/50 °C/min heating/cooling rate.

Structural analysis, purity and phase transformation were analyzed by X-ray diffraction (XRD, PANalytical, Netherlands, 40 kV, 30 mA, Cu *Kα* 1: *λ* = 1.54056 Å, step: 0.02°). The morphological and elemental mapping of the fabricated ceramics were observed by a field-emission scanning electron microscope (FE-SEM, FEI Quanta 200, Hillsboro, OR, USA). Later on, the fabricated ceramics were polished to a thickness of ~0.7–0.9 mm for the ferroelectric, electric, piezoelectric and dielectric measurements. Silver paste was coated on both surfaces of the disks to work as an electrode. The polarization versus the electric field loops (*P-E* loops) were recorded by an aixACC TF Analyzer 1000 (ferroelectric tester) at the fix frequency (1 Hz) at room temperature. Ceramic samples of all the compositions were fully poled at a temperature of 150 °C, with a high *dc* electric field of 140 kV/cm, followed by the measurement of the piezoelectric coefficient (*d_33_*) at 60 Hz by IAAS ZJ-30, Institute of Acoustics of CAS, Beijing, China (piezoelectric tester) at room temperature. The thermal depoling of the ceramics was checked by annealing the poled ceramics at a variable 20–825 °C temperature range for 30 min, and then the *d_33_* values of the annealed samples were measured at room temperature. The dielectric constant (*ε_r_*), dielectric loss and impedance versus frequency curves were analyzed using an HP4284A precision impedance analyzer (Hewlett-Packard, Palo Alto, CA, USA). The dielectric constant (*ε_r_*) versus the temperature curves were measured using an LCR analyzer (HP4980A, Agilent, Santa Clara, CA, USA) attached to a computer with a furnace. 

## 4. Conclusions

Pseudo-tetragonal boundary phased CaBi_4_Ti_3.98_ (Nb_0.5_Fe_0.5_)_0.02_O_15_:*x*wt%MnO_2_ (CBTNF:*x*Mn), with *x* = 0–0.20, ceramics are engineered to inspect the ferroelectric, electric, dielectric and temperature-dependent piezoelectric response. CBTNF:0.15Mn ceramic has shown the best performance among all compositions, with the highest *P_S_* value of 15.89 µC/cm^2^, *W_st_* of ~1.82 J/cm^3^, *ƞ* of ~51%, improved *d_33_* of 27.1 pC/N and impedance of ~ 10^9^ Ω at room temperature. A high *T_C_* of ~766 °C is the main reason for the thermally stable piezoelectric performance of CBTNF:0.15Mn ceramic, even at 600 °C, with just 0.8% *d_33_* loss (25 pC/N). A low dielectric loss < 4%, even at a high temperature of 700 °C, confirms the successful intercalation of multi-valence Mn ions at the A and B vacant lattice sites of CBTNF, resulting in an improvement in the multifunctional properties. The achieved outcomes confirm the ability of the engineered ceramic to be employed in energy-storage and high-temperature piezoelectric applications.

## Data Availability

All the measured or calculated data is available within the manuscript.
